# Development of Novel Therapeutic Agents by Inhibition of Oncogenic MicroRNAs

**DOI:** 10.3390/ijms19010065

**Published:** 2017-12-27

**Authors:** Dinh-Duc Nguyen, Suhwan Chang

**Affiliations:** Department of Biomedical Sciences, University of Ulsan College of Medicine, Asan Medical Center, Seoul 05505, Korea; michaelnguyen1986@gmail.com

**Keywords:** antimiR, antagomiR, miRNA-sponge, oncomiR, CRISPR/Cas9, cancer therapeutics

## Abstract

MicroRNAs (miRs, miRNAs) are regulatory small noncoding RNAs, with their roles already confirmed to be important for post-transcriptional regulation of gene expression affecting cell physiology and disease development. Upregulation of a cancer-causing miRNA, known as oncogenic miRNA, has been found in many types of cancers and, therefore, represents a potential new class of targets for therapeutic inhibition. Several strategies have been developed in recent years to inhibit oncogenic miRNAs. Among them is a direct approach that targets mature oncogenic miRNA with an antisense sequence known as antimiR, which could be an oligonucleotide or miRNA sponge. In contrast, an indirect approach is to block the biogenesis of miRNA by genome editing using the CRISPR/Cas9 system or a small molecule inhibitor. The development of these inhibitors is straightforward but involves significant scientific and therapeutic challenges that need to be resolved. In this review, we summarize recent relevant studies on the development of miRNA inhibitors against cancer.

## 1. Introduction

**Figure 1:**
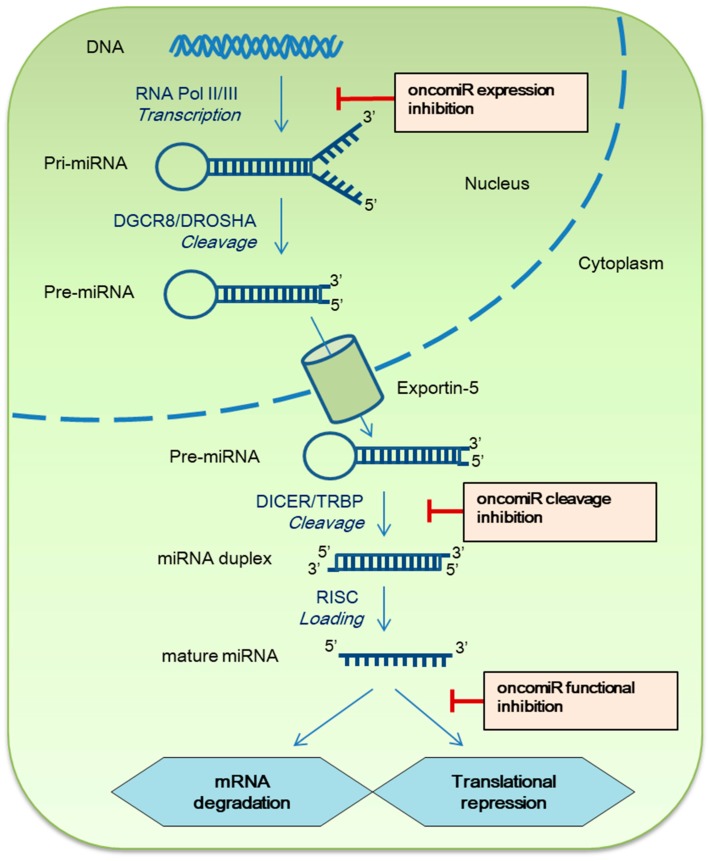
miRNA biogenesis pathway and strategies to inhibit oncomiRs in cancer. The red T bar indicates steps of developing inhibitors for oncogenic micromiRs.

Cancer has been the leading cause of death and a major health problem worldwide for many years; basically, it results from out-of-control cell proliferation. Traditionally, several key proteins have been identified and found to affect signaling pathways regulating cell cycle progression, apoptosis, and gene transcription in various types of cancers [1,2]. Therefore, for years, researchers have been focused on these kinds of proteins as targets for cancer therapies. Lately, alternative approaches to the expression regulation of cancer genes are arousing wide interest with the discovery of noncoding RNA, known as microRNA (miRNA, miR). miRNAs were first identified as small noncoding RNAs that regulate the timing of development in *Caenorhabditis elegans* [3]. miRNAs are 18–24 nucleotides long, single stranded, endogenous noncoding RNA molecules that are natively synthesized in the cell. These short miRNAs can negatively regulate gene expression by complementary binding to the 3′-untranslated region (3′-UTR) of target mRNAs. Rarely, miRNAs control their targets via complementary 5′-UTR secondary structures. This way, they maintain stability of the mRNA of its target genes [4]. The miRNA biogenesis mechanism has been coherently investigated in many studies with the functional diversity of putative target genes [5,6,7]. In brief, miRNA precursors are transcribed from the genome in the nucleus. Subsequently, the long pri-miRNA is generated by the DGCR8–Drosha complex, to produce a 60- to 70-nucleotide precursor miRNA, or pre-miRNA. The pre-miRNA is exported to the cytoplasm via exportin 5 and further cleaved by the Dicer complex into the mature form of miRNA. The mature miRNA is then loaded onto the Argonaute protein, forming a miRNA–protein complex known as the RNA-induced silencing complex (RISC; or microRNA ribonucleoprotein complex; Figure 1). Afterwards, it binds to mRNA and exerts its function of mRNA degradation or translational repression. To date, a huge number of miRNAs has been found and this information is stored in several miRNA databases such as miRbase [8], microRNA [9], or TargetScan [10].

Known as master regulators in the cell, miRNAs are involved in almost all the cellular processes in both normal and pathological conditions including differentiation, proliferation, and migration [11,12]. Statistical studies using genome-wide alignments suggest that roughly 60% of all human 3′-UTRs are predicted to be regulated by miRNAs via Watson–Crick complementarity [13]. Changes in the miRNA expression level to an abnormal state can cause rapid and adaptive changes in gene expression, which can be the cause of various diseases [7,14,15,16,17].

Since the miRNA dysregulation in cancer was first reported in 2002 [18], many studies have been published to reveal miRNAs’ function in carcinogenesis. Now it is widely accepted that the miRNA dysregulation controls cancer development by affecting cell proliferation, apoptosis, migration, and invasion [19]. Notably, the identified cancer-associated miRNAs are diverse and specific for different tissues and cancer types, suggesting that they are potential biomarkers for diagnosis and therapeutic targets [20]. The failure of balanced expression of miRNA in carcinogenesis includes upregulated oncogenic miRNAs (oncomiRs) or downregulated tumor-suppressive miRNAs [20,21]. These key miRNAs have accelerated the development of several approaches to probing miRNAs and analyzing functions in cell culture and in animal models.

This review paper summarizes recent relevant research on the development of oncomiR inhibitors for cancer therapy.

## 2. OncomiRs

Overexpression of oncomiRs have been observed in various human cancers [18,19]. Furthermore, studies have revealed that these miRNAs can function as oncogenes via expression regulation [19]. The regulatory functions of miRNA usually affect its target by the downregulation of expression and play a crucial role in the onset and progression of human cancer. The effect of functional miRNA on its targets is mediated by the interaction of oncomiR with the 3′-UTR and repression of the expression of important cancer-related genes (Table 1). Accumulating evidence validates miRNAs as oncomiRs in the case of their binding to tumor suppressor RNA and downregulation of its expression. Therefore, overexpression of an oncomiR significantly promotes oncogenic properties such as proliferation, migration, and invasion.

Among the oncomiRs identified so far, miR-21 is typical because of its common involvement in most cancer cell lines and tissues—including glioblastoma, breast, colorectal, lung, pancreas, skin, liver, gastric, cervical, and thyroid—as well as in various lymphatic and hematopoietic cancers and neuroblastoma [14]. This oncomiR is a representative example of a single miRNA that targets multiple oncogenic signaling cascades and causes global dysregulation of gene expression networks in cancer cells [28]. A high level of miR-21 expression has been found to target a variety of essential tumor suppressors such as phosphatase and tensin homolog (PTEN) [27,30], PDCD4 [31], RECK [32], and TPM1 [29]; this action facilitates cell proliferation, survival, and metastasis, as well as the acquisition of a chemoresistant phenotype [28].

Another oncomiR, miR-155, is known to be epigenetically controlled by BRCA1 [55]. This oncomiR is found to be overexpressed in breast, ovarian, and lung cancers and often correlates with poor prognosis [37,56]. Recently, some investigators exploited miR-155 as a biomarker and suggested that it may be a potential target in the treatment of B-cell cancers [37]. miR-155 directly regulates some targets; for example, genes *SHIP1* and *C/EBPβ*. Overexpression of miR-155 results in the downregulation of these genes and thus blocks B-cell differentiation and improves cell survival owing to the activation of PI3K–Akt and MAPK pathways [39,40]. In addition, *FADD* and *Ripk1*, which encode a death domain, have been identified as target genes of miR-155, and the targeting of these transcripts may lead to antiapoptotic effects [38]. The expression of miR-155 was also found to be increased in glioma. Further analysis revealed that miR-155 negatively correlates with caudal-type homeobox 1 protein (CDX1) expression in glioma tissues and promotes the progression of tumor formation and poor overall survival [36].

Similarly, a tumor suppressor protein could be a target of other oncomiRs. For example, high expression of miR-10b reduces Krüppel-like factor 4 (KLF4) tumor suppressor expression levels, which are reported to suppress esophageal cancer cell migration and invasion [24]. One of the most frequently mutated tumor suppressors in human cancer—PTEN—is negatively regulated by miR-1908 in glioblastoma cells [53], thus causing an increase in proliferation, migration, and invasion.

**Figure 2:**
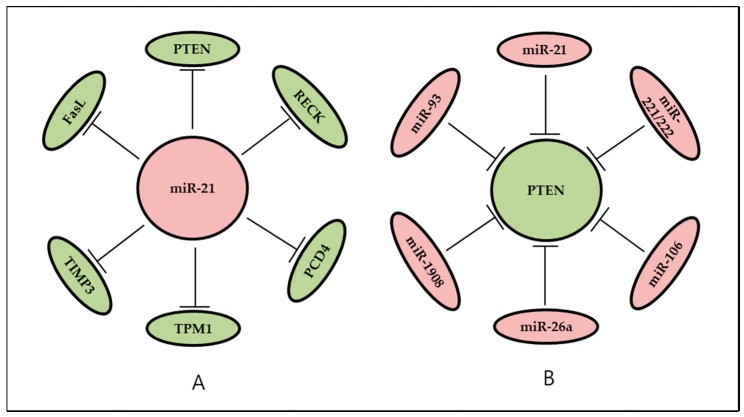
One oncomiR may regulate many genes as its targets, whereas one gene may be targeted by many oncomiRs. (**A**) miR-21 is known as a oncomiR targeting multiple genes simultaneously; (**B**) tumor suppressor PTEN is negatively regulated by several oncomiRs. T bars indicate the repression of target expression by miRNA.

The relations between oncomiRs and their targets may not be only one-to-one but also more complicated. If some target genes share seven to eight 5′ nucleotides of miRNA that are crucial for its target recognition (known as a “seed sequence”), then this situation allows one oncomiR to regulate many genes simultaneously; conversely, one gene may be targeted by many oncomiRs (Figure 2). Thus, it is reasonable to assume that a combination of different oncomiRs may regulate multiple targets as a network and be involved in cancer. Hashimoto et al. [54] analyzed two miRNA combinations, miR-224/452 and miR-181/340. The results showed that overexpression of both miRNA combinations dramatically downregulates their target genes, *DPYSL2/KRAS* and *KRAS/MECP2*, respectively, and decreases cell proliferation. Two other studies proved that downregulation of PTEN by miR-221/222 and miR-106b/miR-93 increases the phosphorylation of Akt. Similarly, the overexpression of miR-221/222 and miR-106b/miR-93 oncomiRs promotes breast cancer cell proliferation, migration, and invasion by targeting the PTEN–Akt pathway [33,46]. As a final example, Park et al. [57] demonstrated that miR-9/miR-21/miR-155 are significantly overexpressed in cervical cancer. In that study, the authors suggested that this microRNA combination as a biomarker candidate for diagnosis and management of precancer patients.

These studies taken together establish specific miRNAs as oncomiRs and imply that an increase in miRNA expression may directly result in the development of cancerous cells that causes the onset and/or maintenance of cancer.

## 3. OncomiR-Targeting Strategies

The influence of oncomiRs have made them attractive targets for cancer therapeutics. For the oncogenic subset of miRNAs, a knockdown of the expression level or blocking the function is believed to be a promising strategy for cancer treatment. The recent explosion in miRNA research has accelerated the development of methods for inhibiting oncomiRs in cancer. These techniques include miRNA inhibitors and oligomers, which can block the functional miRNAs, and are made of DNA or DNA analogs, locked nucleic acids (LNAs), peptide nucleic acids (PNAs), morpholino oligos, miRNA sponges, and other small-molecule inhibitors. Moreover, an oncomiR knockdown can be achieved by inhibiting miRNA biogenesis via suppression of transcription.

Here, we highlight recent research on (and approaches to) miRNA inhibition in cancer, which differ depending on the particular target miRNA and status. Such strategies can be categorized by direct inhibition of single or multiple oncomiRs or the blockade of a miRNA production pathway (Figure 1). First, we will summarize and introduce four main strategies to inhibit an oncomiR that have been developed in recent years.

### 3.1. Targeting an OncomiRs by Specifically Inhibiting Their Mature Form

The widely used loss-of-function strategy to study miRNA function is the use of chemically modified antisense oligonucleotides to knock down specific functional miRNA directly. This antisense oligonucleotide forms a DNA:RNA duplex structure through complementary pairing to target miRNA. This antimiR (or antagomiR) could be an analog or oligonucleotide mimic that is chemically modified to increase its affinity and specificity. Obviously, the treatment of selected miRNAs with antagomiR causes alterations in miRNA-regulated expression of target genes.

**Figure 3:**
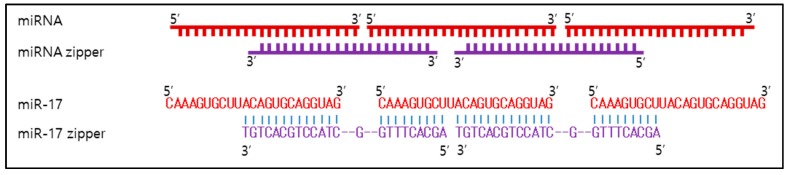
A schematic of a small RNA zipper and its example, which was designed to inhibit miR-17 [69]. A nucleotide gap was inserted between two miRNA molecules and leaves a space for a stable structure, linked to its mature miRNA target sequence.

LNA is typically a modified oligonucleotide and has emerged as a potential therapeutic option for targeting microRNAs. It is known to be effective in functional inhibition of miRNAs with high affinity, low toxicity, high specificity, and stability in vivo [58,59,60]. This molecule contains one or more 2′-*O*,4′-*C*-methylene-linked bicyclic ribonucleoside monomers [61]. LNA has been confirmed to induce A-type (RNA-like) duplex conformation. For this reason, it became a promising choice for targeting miRNAs by antisense-based gene silencing [58,62]. As the first example, LNA-anti-miR-21 was developed and used to verify the functional inhibition of miR-21 in different types of cancer cells, including colorectal [63,64,65], hepatocellular carcinoma, and glioblastoma [66,67]. Treatment with anti-miR-21 LNA results in a significantly decreased level of miR-21 and changes in the expression of its targets [62,63,64,66]. Consequently, it causes robust induction of apoptosis and inhibition of cell growth and of migration. In another study, Dehkordi et al. [68] developed a potent and specific LNA inhibitor of oncogenic miR-222. They found that B-cell chronic lymphocytic leukemia (B-CLL) cell viability is gradually decreased over time after the inhibition of miR-222; they showed that the viability of LNA-anti-miR222–transfected cells was <47% of untreated cells at 72 h post-transfection. A small LNA, termed small RNA zipper, has been developed to inhibit miRNA by generating a DNA–RNA duplex through a complementary interaction with high affinity, specificity, and stability [69]. The small RNA zipper is a small LNA in which half of its sequence is complementary to the 5′-end of the target miRNA, and the other half is complementary to the 3′-end. All individual mature miRNAs can be connected end to end via the small RNA zipper following the zipper strategy (Figure 3) [69]. For targeting miR-221 and miR-17, a concentration of 30–50 nM of small RNA zippers achieves a 70–90% knockdown of miRNA in human breast cancer cell lines [69]. The miR-221 zipper shows an ability to rescue the expression of target genes of miR-221 and reverse the oncogenic function. Moreover, it attenuates doxorubicin resistance with higher efficacy than anti-miR-221 does in human breast cancer cells.

Another class of DNA mimic compounds is named peptide nucleic acids (PNAs) [70] and is an effective artificial agent for targeting miRNA. In a PNA, the deoxyribose phosphate backbone is replaced by a polyamide chain of *N*-(2-aminoethyl)-glycine units [71,72]. PNAs have been proven to have stronger affinity and greater specificity for DNA or RNA than do natural nucleic acids. They are also resistant to nucleases, which is an advantageous characteristic for the miRNA inhibitor that will be exposed to serum and cellular nucleases [73]. Using a PNA as an oncomiR inhibitor, Brognara et al. [74] analyzed the potential effects of anti-miR-221 PNA on the growth of breast cancer MDA-MB-231 cells. Targeting miR-221 by a conjugated PNA in other cancer cells resulted in decreasing levels of miR-221 and upregulation of *p27Kip1* expression at both mRNA and protein levels [73]. In another study, this PNA also inhibited miR-221 in human glioma cells, thereby promoting miR-221 target genes including *p27Kip1* and *TIMP3* [75] and inducing apoptosis [76]. Lastly, Amato et al. designed and synthesized PNAs to inhibit oncogenic miR-509-3p; 3p means the mature miRNA released from the 3′ arm of the pre-miR-509 hairpin structure for distinguishing it from the 5′ end. Researchers have confirmed the ability of the negatively charged PNA1 and positively charged PNA2 to bind to their target miRNA by forming stable miRNA–PNA heteroduplexes [77]. After further investigation, they demonstrated that the activity of miR-509-3p can be inhibited even by means of a PNA as short as seven bases long, targeting the seed region of the miRNA exclusively [78].

### 3.2. Targeting OncomiR by Small Molecules

Until now, miRNA-targeting agents used in preclinical and clinical studies have lacked delivery efficacy and good pharmacodynamic or pharmacokinetic properties. The “lock and key” mechanism is the main idea for screening small molecules for miRNA inhibitors for drug development. Drug-like characteristics of a small molecule are advantageous for resolving the problems mentioned above. Moreover, the secondary structure of pre-miRNA contains a narrow groove, which a positively charged compound can easily target at nanomolar (nM) binding affinity, making it a druggable candidate [79]. Besides, the resolved structures of major proteins involved in miRNA synthesis, as well as the structure of a miRNA–protein complex, enable structure-based approaches using small molecules to inhibit oncomiR specifically [80,81].

Generally, the approach to designing a specific small molecule targeting miRNA falls into several main categories. First, researchers conduct high-throughput screening of libraries of candidate compounds and identify small molecules that are known to be able to interact with the enzymes involved in miRNA biogenesis [82]. There have been several approaches to building a platform for this high-throughput screening. For example, Daniel A. Lorenz et al. [82] reported a new platform, cat-ELISA, for high-throughput screening of RNA−small molecule interactions. Nonetheless, they did not proceed to the discovery of RNA-specific ligands [83]. The luciferase reporter system has also been widely used for the high-throughput screening of small molecules against oncomiRs [84,85,86]. Gumireddy et al. [84] selected miR-21 as a target miRNA for a luciferase reporter system and screened more than 1000 small organic molecules. They finally identified azobenzene-2 as a specific and effective inhibitor of miR-21 expression; however, updates on further research have not been published.

Alternatively, investigators can focus on a small molecule that is predicted to be able to interact with RNA via sequence and structure analysis. Inforna is a platform for the sequence-based design of small molecules targeting RNAs [87]. However, a database has been generated containing 1936 RNA motif–small molecule interactions, including 244 unique small molecules and 1331 motifs, which were collected and updated from all known RNA motif–small molecule binding data so far [87]. Vo et al. [88] focused on two RNA-binding motifs of small molecules for pre-miR-372 and pre-miR-373, to develop a miRNA inhibitor for gastric cancer cells. Two conjugation reactions—of an RNA-binding motif in a small molecule with high affinity for pre-miRNAs—disrupted the splicing process of oncogenic miRNA-372 and -373 [88]. Finally, a combination of bioinformatics and a high-throughput fluorescence–based screening system is another approach. Recently, a study revealed that inhibition of Dicer cleavage sites in pre-miRNAs by means of a small molecule abrogates miR-544 production, resulting in sensitization of breast cancer cells to hypoxic stress [89]. Expanding similar studies on targeting of miRNA with small-molecule libraries and a protein–RNA structure database holds great promise for identifying a variety of novel agents capable of inhibiting oncomiR production.

### 3.3. Multiple-OncomiR Targeting by a miRNA Sponge

The miRNA upregulation in tumors affects not only a single oncomiR but happens to multiple miRNAs, reflecting their combined biological effects. Therefore, the crucial antimiRNA strategy needs to have a requirement for cotargeting different miRNAs that often belong to the same miRNA family. On this basis, a DNA construct has been introduced as a potential tool for developing a multi-miRNA loss-of-function system applicable to both in vitro and in vivo studies.

One of the most widely known constructs of this sort is a miRNA sponge, which has the ability to capture multiple miRNAs. It was initially introduced in 2007 [90] and achieved stable inhibition of miRNAs in cancer cells, as well as in transgenic animals [91]. A miRNA sponge is a DNA construct(s) that contains artificially designed miRNA-binding sites in the 3′-UTR of a nontoxic gene. The expression of the miRNA sponge with specific miRNA-binding sites can tie up those endogenous miRNAs, essentially depleting the cell of the target miRNAs. A miRNA sponge can achieve stable inhibition, as well as inducible/tissue-specific inhibition, of target miRNAs in vitro or in vivo [92].

Recently, Barta et al. [93] introduced a web-based tool for the generation and in silico testing of miRNA sponges, named miRNAsong. This tool generates a miRNA sponge construct for specific miRNAs or miRNA families/clusters and tests it for potential binding of the miRNAs in selected organisms. The miRNAsong software contains 35,528 miRNA sequences, allowing for the design of sponge constructs before actual synthesis. Zhou et al. [94] constructed an miR-221/222 sponge and transfected it into CAL27 and HSC6 OSCC carcinoma cells. The sponge triggered apoptosis and a reduction in cell proliferation and invasion through miR-221/222 inhibition and upregulation of PTEN. Moreover, recent papers have shown inhibition of multiple oncomiRs of the miR-106a–363 cluster by a miRNA sponge in Ewing sarcoma [95] and targeting of the miR-183/-96/-182 cluster in breast cancer [96].

In our laboratory, Jung et al. [97] demonstrated a multipotent miRNA sponge as a useful tool to examine the functional effect of simultaneous inhibition of multiple miRNAs and proposed its therapeutic potential. To achieve an effective knockdown, cotargeting of miR-21/155/221/222 was tested. In this multipotent miRNA sponge, perfect or bulged-matched miRNA-binding sites (MBS) were introduced as tandem repeats, ranging from one to five. A luciferase reporter assay showed that the multi-potent miRNA sponge efficiently inhibited four miRNAs in breast and pancreatic cancer cells. Furthermore, an inducible version of the multipotent miRNA sponge was stably expressed in cancer cells and showed an effective reduction of the four target miRNAs with an increased target protein level. Consequently, we found that the expression of a miRNA sponge sensitizes cells to a cancer drug and attenuates cell migratory activity.

### 3.4. Targeting the OncomiR Synthesis Pathway by Genome or RNA Editing

**Figure 4:**
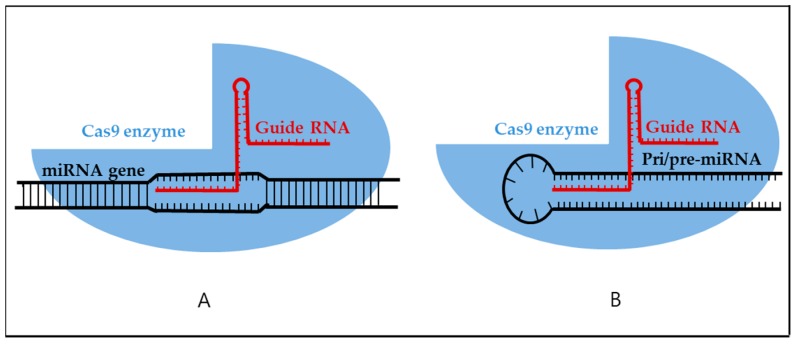
The applications of CRISPR/Cas9 editing systems to miRNA targeting. These two strategies include (**A**) a classical method by DNA genome editing or (**B**) a direct method of editing the oncomiR precursor.

Understanding the miRNA biogenesis pathway has opened a new road to a knockdown of oncomiR. This strategy can be undertaken by genome editing in or near a specific site of the precursor miRNA or DNA location (Figure 4) resulting in deficiency of mature miRNA and its function as an oncomiR.

The CRISPR/Cas9 system has rapidly emerged as a state-of-the-art genome-editing tool [98]. Application of CRISPR/Cas9 to a specific miRNA is a novel approach to therapeutic inhibition. In a recent report, researchers tested this idea by disrupting miRNA genes by CRISPR/Cas9 in a macrophage cell line [99]. One year later, application of this system to the oncomiR knockdown was developed in cancer cells [100]. In this study, CRISPR/Cas9 was employed to target miR-17/miR-200c/miR-141 loci, and the results showed decreased mature miRNA levels accompanied with low off-target effects in HCT116 and HT-29 human colon cancer cell lines. Moreover, the miRNA knockdown phenotype caused by the CRISPR/Cas9 editing can be stably maintained in both in vitro and in vivo models for up to 30 days. However, Hou et al. [101] successfully constructed two miR-21 lentiviral CRISPR/Cas9 guide RNA vectors to repress miR-21 function in ovarian cancer cell lines. The data showed that disruption of pre-miR-21 leads to upregulation of miR-21 target genes, *PDCD4* and *SPRY2*. Via disruption of the miR-21 precursor, the authors successfully inhibited proliferation, migration, and invasion of ovarian cancer cell lines. Another group reported an approach to targeting miR-130a using the CRISPR/Cas9 system in the MCF7 breast cancer cell line [102]. The results revealed that the expression of miR-130a-5p, but not miR130a-3p, was successfully downregulated by CRISPR/Cas9 targeting. OncomiR-10b, a key regulator of tumor growth and survival in glioma cells, was also eliminated by CRISPR/Cas9 in a brain cancer cell line [103]. The loss-of-function mutation blocked the escape of proliferative clones of glioblastoma and induced cell death.

Recently, a report showed applications of the CRISPR/Cas9 system within the RNA level editing as a molecular tool for editing noncoding RNAs in human cancer cells, including miRNA [104]. This application may provide an excellent way to cure cancer by noncoding RNA interference. Additionally, the off-target effects and the corresponding solutions, as well as the challenges for the novel uses of this technique, were evaluated and discussed elsewhere [104].

Genome editing as miRNA inhibition therapy not only targets a single miRNA but also disrupts multiple miRNAs. Recently, Narayanan et al. [105] performed a global screening of 45 mutations in 10 miRNA genes, analyzing the impact of the CRISPR/Cas9 mutagenesis strategy on the processing of each miRNA both in silico and in vivo. The results indicate that 99% of CRISPR/Cas9 mutations alter critical sequences within each hairpin pri-miRNA structure; these mutations impair recognition by the miRNA biogenesis machinery, thus preventing the miRNA family expression in vivo. These up-to-date studies collectively suggest that the CRISPR/Cas9 system can be adopted easily, stably, and cheaply to engineer and inhibit miRNA function for cancer-therapeutic purposes.

## 4. Challenges

Many examples of oncomiR biomarkers and potential therapeutic targets have been presented so far. Nonetheless, the dynamic changes of many oncomiRs and their targets generate multiple regulatory mechanisms that make focusing on a single target insufficient for clinical application. All selected stories above serve to highlight the roles and strategies for inhibition of oncomiRs in cancer research and therapy. Each strategy has its own advantages but faces many limitations that have to be overcome at the same time. For example, in the oligonucleotide- and peptide-based therapy, one advantage is that there are no requirements for additional materials; however, the main difficulty is that nucleic acids are easily broken down by endonucleases, are immunogenic in the bloodstream, and are difficult to absorb by the cell, making systemic delivery of naked molecules ineffective [106]. Even though chemical modifications or conjugation (e.g., to a nanoparticle) can protect antisense oligonucleotides from degradation, challenges related to the toxicity of materials and the high effective dose are still present and need to be addressed [107]. Therefore, effective methods of delivery of miRNA to humans need to be devised for a breakthrough in the field of tissue damage research and safety. As an alternative strategy, multiple oncomiRs may be inhibited by CRISPR/Cas9 via the genome engineering technology. Nevertheless, this technique is on the long road of development for use in the clinic, where off-target-free, precise editing of oncomiR genes is expected.

Furthermore, the miRNAs in cancers are diverse and need to be confirmed specifically. Nonetheless, the use of miRNAs as biomarkers for targeting and diagnostics is certainly important in cancer. Besides, it is important to document various miRNA profiles because of race, gender, and age. miRNA profiles may also change because of the effects of chemotherapy, radiation, or surgical treatment: the mechanism of miRNAs’ upregulation needs to be characterized.

## 5. Clinical Research Examples of AntimiR Therapeutics in Cancer

Certainly, miRNA targeting is a new discipline of general interest and needs to be studied regarding treatment of cancer and other diseases. Table 2 summarizes several examples of therapeutic molecules against miRNAs from recent studies.

OncomiR-10b is known to initiate robust invasion and metastasis by multiple-gene targeting in glioblastoma and esophageal and breast cancers (Table 1) [24,25,26]. These studies indicate that miR-10b may well have a causal role in cancer development and point to the need to inhibit its function in those types of cancer. Regulus Therapeutics is currently developing an miR-10b antisense oligonucleotide (ASO)-based inhibitor as a potential miRNA-inhibitory treatment of cancer [108]. They obtained promising data after systemic administration of the miR-10b ASO at a preclinical stage and are looking forward to the first steps in the clinic.

Similarly, Regulus Therapeutics is developing ASO-based antimiR for two oncomiRs—miR-21 and miR-221—that were confirmed to negatively regulate the key tumor suppressor PTEN in cancer (Table 1). Thus, the two oncomiRs miR-21 and miR-221 were chosen as promising therapeutic targets for inhibiting the oncogenic phenotype in cancer cell lines and tumor xenografts. In preclinical studies, they demonstrated potent inhibition of miR-21 and miR-221 in vitro and in vivo by means of an antisense oligonucleotide [109,110]. Moreover, it was reported that miR-21 upregulation promotes fibrosis in the kidneys of animal models [114]; these findings support the company’s project to develop the miR-21 ASO as well, named RG-012. Recently, they reported that RG-012 received an orphan drug status from the U.S. Food and Drug Administration and European Commission as a therapeutic in development for the treatment of Alport syndrome.

Another well-studied oncomiR associated with poor prognosis in multiple cancers is miR-155 [36,37,38,39,40]. An optimized LNA-modified oligonucleotide inhibitor of miR-155, designated as MRG-106, is under development by miRagen Therapeutics [115]. So far, MRG-106 is being evaluated in patients with cutaneous T-cell lymphoma at the I stage [111].

Santaris Pharma is developing an antimiR-122 named miravirsen, which is an LNA-modified molecule. The updated results revealed no long-term safety issues among 27 miravirsen-treated patients [112]; there was a prolonged decrease in plasma miR-122 levels in patients dosed with miravirsen but the plasma levels of other miRNAs were not significantly affected by antagonizing miR-122 [113]. The investigation is now in phase II. This antimiR therapeutic may also be an effective and safe treatment strategy against hepatitis C virus (HCV) infection in the future. Although this review focuses on targeting oncomiRs, we include the clinical development of miR-122 inhibitor for HCV infections in order to study the potential of therapeutics targeting miRNA in cancer. Many antimiR therapeutic agents are now in preclinical and clinical development. Given their leading edge in druggable use, oligonucleotide agents have several advantages over other modalities because of easy modification and specification.

## 6. *N*-Acetylgalactosamine (GalNac) Conjugation Enhancing the Potency of ASO Therapeutics

As mentioned above, the effective delivery of an active oligonucleotide to its site of action is the key challenge in realizing the full potential of oligonucleotide therapeutics. A ligand–oligonucleotide conjugate is one of the delivery methods that has advantages for transport of an oligonucleotide or peptide for therapeutic purposes. It offers the capacity for selective delivery to specific cells or tissues via receptor-mediated mechanisms if the ligand is conjugated directly to the oligonucleotide specific to a certain target. GalNac, or triantennary *N*-acetylgalactosamine, is the major breakthrough in oligonucleotide therapeutic delivery, which represents a high-affinity ligand for the hepatocyte-specific asialoglycoprotein receptor (ASGPR) and enhances the potency of ASOs 6- to 10-fold in mouse liver [116]. The excellent properties of GalNac have led to compelling successes in the development of oligonucleotide therapeutics. Several oligonucleotide modalities are undergoing pivotal clinical studies, followed by a blooming pipeline in the preclinical stage [117].

The application of antimiR in cancer is now at an early stage, and many of these studies are in a preclinical phase. Although the conjugate approach is still in its infancy, it seems to offer a promising path forward for oligonucleotide therapeutics.

## 7. Concluding Remarks

OncomiRs have been demonstrated to play a causal role in the onset and progression of human cancer. Since their discovery and characterization, the number of oncomiRs deposited in databases has increased greatly, and studies on oncomiRs have confirmed the high complexity of the regulatory networks involved. In this review, we compiled the recent reports on oncomiRs and focused on the most promising examples that may lead to oncomiR therapeutics. These include a single miRNA or multiplex miRNAs with LNA/PNA modification, a microRNA sponge to block functional oncomiR, or a specific knockdown of an oncomiR by genome editing. Improvements in the stability of antimiR candidates and in drug delivery systems along with the investigation of off-target effects are several prerequisites for successful translation of antioncomiR therapeutics from the bench to the bedside.

## Figures and Tables

**Table 1:** Oncogenic microRNAs (miRNAs) in cancers.

OncomiR	Targets	Cancer Type	Ref.
miR-9	E-cadherin, LIFR	Breast cancer	[22,23]
miR-10b	KLF4, HOXD10, TP53, FOXO3, CYLD, PAX6, PTCH1, NOTCH1,	Glioblastoma, esophageal, breast cancer	[24,25,26]
miR-21	PTEN, PDCD4, RECK, TPM1	Glioblastoma, breast, colorectal, lung, pancreas, liver, gastric, cervical, and hematopoietic cancer	[27,28,29,30,31,32]
miR-106b/93	PTEN/Akt pathway	Breast cancer	[33]
miR-125b	P53	Lung cancer	[34]
miR-130a	CRMP4	Gastric cancer	[35]
miR-155	SHIP1, PI3K, FADD, CDX1, C/EBPβ	B-cell cancers, glioma	[36,37,38,39,40]
miR-181a	PRKCD, Bim	Cervical, breast cancer	[41,42]
miR-200s	ZEB1, ZEB2, SIP1	Breast, ovarian cancer	[43,44]
miR-210-3p	SOCS1, TNIP1, NF-κB	Pancreatic cancer	[45]
miR-221/222	PTEN	Breast cancer	[46]
miR-335	Rb1, Bcl-w	Ovarian cancer	[47,48]
miR-498	BRCA1	Breast cancer	[49]
miR-504	P53, CDK6	HSCC, neuroblastoma	[50,51]
miR-1810	PDCD4	Colorectal cancer	[52]
miR-1908 miR-224/452 miR-181/340	PTEN DPYSL2/KRAS KRAS/MECP2	Glioblastoma Gastric cancer	[53] [54]

**Table 2:** Recent examples of antimiR therapeutics.

microRNA	Inhibitor Agent	Type of Disease	Investigation Status	Company/Ref
miR-10b	ASO	Glioblastoma	Preclinical	Regulus Therapeutics [108]
miR-21	ASO	HCC, fibrosis	Preclinical	Regulus Therapeutics [109,110]
miR-155	LNA-modified	T cell lymphoma and mycosis fungoides	Phase I	miRagen Therapeutics [111]
miR-221	ASO	Pancreatic carcinoma	Preclinical	Regulus Therapeutics [110]
miR-122	LNA-modified	HCV	Phase II	Santaris Pharma [112,113]

HCC: Hepatocellular Carcinoma; ASO: antisense oligonucleotide inhibitor; LNA: locked nucleic acids; HCV: hepatitis C virus.

## Abbreviations

3′-UTR	3′-untranslated region
ASO	antisense oligonucleotide inhibitor
Bcl-w	B-cell lymphoma-like protein 2
Bim	B-cell lymphoma-like protein 11
BRCA1	breast cancer type 1 susceptibility protein
C/EBPβ	CCAAT/enhancer-binding protein beta
Cat-ELISA	catalytic enzyme-linked click chemistry assay
CDX1	caudal-type homeobox 1 protein
CRISPR	clustered regularly interspaced short palindromic repeats
CRMP4	collapsin response mediator protein 4
CYLD	cylindromatosis
FADD	Fas-associated protein with a death domain
FOXO3	forkhead box O3
GalNA	*N*-acetylgalactosamine
HOXD10	homeobox D10
HSCC	hypopharyngeal squamous cell carcinoma
KLF4	Krüppel-like factor 4
LIFR	Leukemia Inhibitory Factor (LIF) receptor alpha
MAPK	mitogen-activated protein kinase
NF-κB	nuclear factor-κB
NOTCH1	neurogenic locus notch homolog protein 1 precursor
PAX6	paired box protein
PDCD4	programmed cell death 4
PI3K	phosphoinositide 3-kinase
PRKCD	protein kinase C delta type
PTCH1	protein patched homolog 1
PTEN	phosphatase and tensin homolog
Rb1	retinoblastoma
RECK	reversion-inducing-cysteine-rich protein with kazal motifs
SHIP1	SH-2 containing inositol 5′-polyphosphatase 1
SIP1	Smad-interacting protein 1
SOCS1	suppressor of cytokine signaling 1
SPRY2	sprouty homolog 2
TNIP1	Tumor necrosis factor, alpha-induced protein 3 (TNFAIP3)-interacting protein 1
TPM1	tropomyosin α-1 chain
TP53/P53	tumor protein p53
ZEB1/ZEB2	zinc finger E-box-binding homeobox 1/-2
